# Supplementary material for: Architecture and evolution of the cis-regulatory system of the echinoderm kirrelL gene
Source: eLife. 2022 Feb 25;11:e72834. doi: 10.7554/eLife.72834 (PMC8903837; doi:10.7554/eLife.72834)
Supplement: Figure 6—source data 2. [file elife-72834-fig6-data2.docx]

**Figure 6 – source data 2**: DNA sequences for CREs validated in this study from *Eucidaris tribuloides (Et-kirrelL), Parastichopus parvimensis (Pp-kirrelL), Patiria miniata (Pm-kirrelL), Acanthaster planci (Aplc-kirrelL), Ophionereis fasciata (Of-kirrelL), and Anneissia japonica (Aj-kirrelL).*

>Et_kirrelL_CRE

TGGGAATTTGCTTTATTGTCAGATATGACATCAAAGATGACATTTCTAGTTAACCAAAACACCAGTGTATACATTGTATATCACTAGATGTCTTGAAATTATGTACTAGACATTTGGTTAACTGGAGTTTGTTTATCATCTTTGATTTCATACGGGACAATAAAACAGATTTCTTAAATGGACTTGGGTCATTTTTTATATTCAGATACTCAATTGATAAATCAGTCGCTCAATCAATACTCCTCTTTACAATGGAACTTTGATATCACGTCATATATCACATCTATGTGAAAAAAAAATACATTTTACTTTTACTTGTCAGCACACTTTTTTCTAGTCATTAAAGTCCAATGGAATTTGTGACGTATATTGATGATGCCGACAGGTTGATTGCCGGCAGATTATGGCGGGAGGTAGAGAGGTGAATTAGGTTTGCATGTTGCTAGTCAACGTGATAGACATATCGTATGCATGCAAGGCAATAACCTTTTATATTTTGAGATTGAAACCAACTTTAAAAGTGTGTGTAACGCTTTTAAGGAAACATAAAATTCAAACATGTATTTACGATTCAAGTTGTTCTCAGTTCTGGTAAGACGGAAATCCCTTGAACTGCTGTTATGACGTCGATATAATTTCAAAAGGGGAAACGTCTTATTCTAAATGTGTAATTTCTACCGACCAAGTACAAACATGATGATTAAATTAATTTATTTTGTTACCATATTTGTTAAACTTCAGCAAAAACAACCAAACGCCCACGCCAGAATCTTCTGGAATGATGTGTGGGTAGGAAAGCAAACTTTCCATCACCCGTTTCCTGTTTTGGTCGAATTGACTTATGACTGTACTATGTTCCCCAATTTAAGGGGGCAACAGACTGACTGAATTTTACTAGGTTTTATTTAGATGTACAGTTGGATAGGGCTACATGACTGATAATGTAAATGAAAAAAAAAATACCTCAAAATTGTTTTCGGGATCGTTGTGGAAATGTCAGTTGTATCAGAATTGAACTCTCACTGTGTGAGGCAAATGTATTTCAAAAGGTTGAGTCTGTTATAATTAAGGTAACATGCATTCTGTCAACAGTATTATCCCCGGTAACTACCTTGCTAAACTCGGGAGTTGTTTACAAGTGGTTCTCTCAATAGAGTCACAGAATTCGAGTCATTTATAGGACACAGAATCTGATTTCCGAATGACTTCGACGTGCTAACATACGATATTTGCGTATGCTAAAAGCCCACGGTTTTCCTGTTGGCTGATACAAAAGAGGAAAACGCCTAAATCTAATAAGAGAACCTAACCATCGCGAGCAGCGCGCTGTACTCAGTTGACATGCTAGTCTGACTTATACACGGATGTTACACATTCCAGAAATATATCACCGTCCGAGGGTGCCTAGTGCGGACTTAAACAGATTTTCACACATATAGTGTTTGCTTGACCAGTGCATATACGTAAAACTCACCTGTAAGTGTTAAATAATACCAAAACTTTACAGACAATGAAAAAAGGCGTTCGGCACCGAGACTAACTAGCCATAACTTGACTCAAGTAGCACGCACTCACGACAACTCACTGTCACACGGACGCATTGCTCGTTGCGGTACAGACTCACTTTCATTCAACGCGGGTACCAGCCACGGCACACAGATCTCATCACTGTCGAGTTTTCAACACTGTGGCAAGAACTTGTACATAAATTATTGTTATTTTTGTCGCAACTGAACTTTTTCGTTAACATTGATTAAGCCGGC

>Pp_kirrelL_CRE

ATATACACGCGATACATAATAAAAGTATCAGTTATATGCCGTCTGAAAACAACAATTAACTTCAGAATGTCTGTAGTCTTCACAGTCAAAAGAGAAAAGAGAAAACGATGGCCTTCTTTCATATAATTGCATTTGTTACAAAATTTACCCACAAGACTAATAACTAAAGTCATCATACATACCGAATTAAACCTCCTCCTCGTGATATTACGAGATCACGTGACATTCTCTGTGAAGACTTCCCGCCTGTTTTCATTGGCTGCTGCAAATGATGCTTAGCCAAGTGTACACAATTATCCATACGATGGATATACAATGATATTCCCTAATGATTATACATGGTATCAAATTCATCGCCTCGCTACCTTTAATGGTAACTTAAATCTTGCCGAAAATAAAATAAAATAAGTCGGCCTATATGCCAATAAAAATACAAGCGAAGCGCATAACTTGCTGGTATCCGTAAATCATTTTCAAACCTGCCTGTATCCTATTAGGTATTACTAGTCTGTGTATAGGGATTCGTTTGCATGTAAAGGTGAGAAGGGAGATAACGACTTAGTGCAAATAAAACAAGGAGAGATAGAGATATAGAGAACTGAGAAACAATGGCTGTTGCTGAGATCTAAATTATGGTCCTTGTCAGATGGAAATATTTAATGGAATTAATTATTCTGCGCTATTTCAATTTACGTTTTTCGTTGATATTCTGGTTCATTTATTCATGTGGGATATACAAAACGACTAATCAAAAGATTTCTGCCAATTATGTTTTTGGAATTATTTATCTCCGAGCAATACACGCGAGAGGAACGGATTAAGCTTTTATCTTCGAATCAATGACATAGACCGCTATATGGTCACAATACCTTAATTTGCATATTTTGTGTTAAATGCGATGACATAGTTTTTATGGTGCCAGTTATTTGACAGGTTTTGATCATAGAATTCTTTCTTTGCTGCAGATGATTATTATTCTTGCTATTGATGATATATCTCGGAACTGGTGATATCATCAATTCTTTACAAAATCAACATTTCTTCATAGTGACGTTTTCGTGCAATTTCCATGTATTCTGTTATCTTTTGATATCGCGTATAGAATTTACCCAAAAAATGTAGCTTTTTTGGGATCCCTCGGTTTCCATTTGTGTTTGTGTCTTTACGAAGAAAATTGGAAGCGTAAATTTTAGTGACAAAATTCCGATGGAAAAGTCTTCACGCTTTTTAAATGTGCGACGAAATATCAACTATATATTTGGAGAAATAATGCAGTATTCAAAACTGGTAAAAACCTTATTTGTTTGTTTTAGAATCTATAAACTTTCTTTCTCTCCTACTAAAATCTTTAAAATTTGATTAACCAAAATTGAAAGAAGTAAATACCTTTCATATCGACTTACAGTAAACGCCTAGGCTTATATACATTTGTCTTTACTTCTTGCAAATCGCATCCACTACAGAAGCCATTCCACTTCATCAGTCATCCAACTATATGACTTGCTTAAATATTTGGCTTCAAGCAATCTCTGTACATGGAAATCCATTTTTTTCTTTTAAAAAACAACTCCAAAGCTGACATTTGAACTTGAATTTAATCTACACTTGGCTTGACAACCAGTTCCTCATTTACTTGATGAAGCGGATGGTTTTGATTAACCTAATCATTCAGTAGCAAGGAAGGGGTGTTGTGTTGATAGCTATGCGGATGGACTAGGAGAAGTACTCGCGCCGTGAATCAACTATTGACTCAGTCAGATCGGCTGTTGCTAATCAAAAGTTTGGTATAATTCTTCAAAGGGGGAAACGGCTCGTTGCAAATGCTGTTGTTTTTCTTCCAAACTGTTTCATGAAGGGCTGATTTCACAGTATTGGATATAGGATTTTAAATTGTTTGAAACGTTCAGAAGGGCCAGTCAGGTCTACATAGGGAATTTATTGCTTAGATGTGGATACATCCAGCAAT

>Pm_kirrelL_CRE

GAGCAACAGAAGTGATGCTAATACAGATCCTTTAGGAATTCGATAAAAACAGTGGAATTAATTAATTAAAAAGAGAATAGTTGTTGAAAATTCAAGATTTTGTTTAACTATTTGACAAAAACAAAAGCAAAACGCGGGCGTTATAGACATTATTTTCATAAAACAACTCAAAATTGTTGTTTCAAAAATAGATTTGAAAATTTGGGTCACACGGCTTTCCATTGTTATCTTATATAGCAACGCCCCCTCTCAGTCCTGTGCATGGATGAAGTTCCCTTTTCCATGCCTAATCGTTTCCAGAAGTATAGCTCACATAGGTATTAGAGAGTGACCCCTCTGTTGTTATTCAAATCGACCAATGAACGTCGACCGATGCCATTTGTATCTGGTTCTATTGGTCAACTGCCTAATTTTGATTGATGTTCCCTTCCACAGCTGTGTCCTCCATTTGTCGCGAGATATGACATTTCCTCTGCCTATACCGGAATCAAAATATGAACCGCCCATACTTCAAGGCCACCAGGGAGGGTTATTGGGTCAAAAATATCAACCGCATAATTATGCTCACTCGTTCCCACCGATCCGTCCAAGGACATGGTAACCTCTAATTTCTGTGAGCTCCCATTGGACAGATTTTGTTGCACGACAACAGATTTTCATTTTGAAAGGTATAATCTGCTGGTTCAAAGTACCATTCGGAAGTTACTTTCATGGAAGGGCATGACTGCAAAACCCGCGAATTGACGGTCATATTTTGTGAAATGTCCCACCCTCAGGTTTGCCGTCGGCGTTTGTCATGATGACGTTCAGTGGTGTCTAGACTCTGACCGCTAAGAAACAAGGATTCGCTGGACATGACTGGTCAGATCTGATCGGTCAGATCTTATTAAAGGAACCCTCTTATTACGCGAATGATGCAGCATTATGGCTTGAAGACGATGTACAATGGTTATCGTTAAAGGAAAGATTATAAAGCACTCGGAGCGGATCCAAAAGTGAGGAATATTATTCAACATGTGGACCAAAATAGAACTTTTTTTCAAAGTCGAACTTGAAGGTTTGCATGGTTAAAAGGAAACCGCGAGTTGAGGATTTTGCGGTAACAGATTCTTCAGGAGACAAACATAGCACACTGCTCGAAACGTTGAGTAAACTCACAAGAGATACACTTGCGGAGCGACCAAAAAAAGGTTTACTTCAGCGAAATAAGTTTCCGTTCGGTAAATTCAGTGACACAGCCCACTACTCGCAGAGCTATATGTGCTGTCTTCGCTTGTGAGGGAAAAGTGTTATCTTCATTTGTGAGAGAAATAAACATCTCTTTGGGTTTAATGTTTTGTTTATGTAAAACGGCTTTTTTACGTTTAAAAAAAAAGGAGGTTTTTCTATGTACCTGTATCGTTTTCATATCCTCCTTATAGATGAATTAACACTGTAGAACATTTTTAAATAATTATATTCAGTGGCCTTGAACAAAGAAGCAAACATTTGACATGATTAACGATGTGTTTTGTACTAGAATGAAGAAAACTTGGCTGAATATCCCCGGGGGAGTGGAGTGAACCAAGGACACGGAAGTACACGACGAGTTCCGGAGGAAAAAATGGTGGTGTTGACGCAACGATTATATTTTCCCTTCTTTCCAAAAGTTTGAAGTTGTATCCACAACAAACCTGCTCCTACAGCCGGCTTTGGGGTTATTTTTACCCCTAGCCAGAGTCCCGTGACATGACCCTCTGACTCCTGAAGGAAACAGACTAATTCGATTAGCCTGGCCCTGGCTGTTTTTCCCCCTTTACTCTCTACCCCCTTTTTGATAGGTCCTAAGGTCTTGACAGGGGGACGGGGCCGGCCTGTCAACCTGTGCCCATGCGTTAATCATCAGCAGTTCCTGAACCGCGTGACGAAGCAGTCGGCTATTGGCTGGTGCGCTCAGCAGGAACCCCATGTACTTGTATCTCCCGGCGTTCTGACTCAAATGTTCCGAAGGTAAGCGCTCGCGCTGTTGCTTTTATAATTCGACTGTCAGACCGAAAGCCCGCCATTTTCACTGACGAATCTGACGGACAAACTTTGCCTTTTCTTCGAAAGAGGATTTGTTATTGTGCAGTTAGAAGTAACCTTAAGATTGATTGACATCGTAGATTGATAAC

>Aplc_kirrelL_CRE

GACAACGCGCAAAATTTGTCGACCCTTTCCAAACGCGTACTGGGTCAACAGATTTCGCGCATTGTGATTGGCCAACGCGTTGCAAACTCTTGGAGGCGCACTATGGTGAAACGCCCGTGTTTATTTTTGAAATGTTCTCCCTTTTTGAGTGCTACTGAATACATTTTGGTTGTTTGGAAGTCTGAAAAATTCTTGGAGATGGTTCTAATAGCTCATATCCCCCTGGTAAAAATGTGGGGAGGATTATAGGCCTATCCCTTCTCCCCGGGGATTTACGCCCATTGTGTAAATGTGAACTCAGGGTTTAAAAGGAGTGATTATTACGGGCATATCTTATTCAACCGTAGATCTGTTGACTCGAAAAGACCCAGGCAGAAACCAACGCGTCATGCAGTTTCTCCAGTATAGTGTTGCCCTTTTTATGCTATAGACTGTCCGCAGAACACATCGTGAACAGTCCAGCCCAAATGCCCGCTTTCTAAAGTTGGAGGTCAAGCAAACTTTCCCCTCTGCTTCCAATGAACACGCTCTGATGAGCTGCGTGCAGGTGTTTGTAAATGATCGTACCGACAGACCCTGTGTTGGTTACGTGGAACTTTACAGATGCGTCCACAATTTGCCAAGCTTTAAAAAGTTTGGGATAATCGAAACCCTGGGGGAGGAATCCCCGGCCCTATATACCAACTGTGAGAGAGTCCCAGGAAAAACGGCTGAGGAAGACCTGTGTGATGAGATCTGTATCACCTCACCACAAAGTTTGAAGCCGACAAAGGAGGGTCATCTTGTCAGTATCCGCCACTGACCTGTTGCCTACGTATAGCCATTTCTCATTAAAGGAACCCCCTGATTGAATGCATAACGCAGCATGGAGTTAAGATAGTGTGATACCACTTGTTGCTTTATCTTTATGAAGGGTGGACAACCGACTTAAGTGACTTACAGAATGAGTAATCAGGGTAGATTAACGTGTCAAGGAAAATAAATTTTAAGTTTGTAAATACGGTACCCCCACACACACACCCACACAGGTTCAAACTTTGGGTATAGAGGTCTAACATTTCCGTGAAAAAAAAATCTTAATTTTACTCGGCCACCTTAGCTTGTTTGTTTCATGAACAAAAAAAAGAAGTGTGAGGAAATTCATCTTTTAAAATACACCTAGAATTTCAATTAAAGCTATCTAAAGGTCTTCAGTTTTCATTTGAAAGTAACTATTTCCCTGCTTTTCTTCAAAGTTGCAGAAAGTGTACTAAATTATTCATTAAAAATGAATGAAATTGGATATATTAAAATTAAGGCCCTGAATATTTTTTTCTGGTATAATCATTTAAAACTGCGAGCTTTCTGATATGATGGTTTAAGTGCTATATGAACGTGACAAAGGTTTATCTAACGGTCATCACTTAGTAAAGGCATTTGGCGGTTGTTTTGTTTTGCTCAGCATTTAATTAATCTTTTCTTTTCTTTTTCAACGCAACTGATATTCTCGCCAGACTAACCTGAAGATATCACTATATCATTCTCGAAGTCTGGAGTGGCCTTGAACAATGACTTATGGCATGTTTTTCTTTAAAAAAAGTCCCTGCCGATTTCCCCCTGAGCGTGTAGCGAACCAAGGATGATGGAAGGAAAGGGCGGGTTCGGAGGGGGGACTTGAATCTTGACGTAACCAGTTTTCCTTCTTTCCCAAAGTTTTGAAGTTGTATCCACAACAACTTTACTTCTTATTCCCGGCGTTGGGGCTATTTTTAACACGAGCCGGTGTCCCATGACATCGGCCTTCTGACTACTGTAGGAAACAGACTAATTCGATTAGCCTTGCCTGGGCTGTTTTTCTCCTTCGCTCGCCCTTTTCTGCTGTGCAGTCCAACAGTCCTGACAAGAGGGCAACGGGGCCGGCCTGTCAACCTGTGCCCATTCGTTAATCATCGCCAGTTCCTGAACCGCGTGACGAAGCAGTCGGCCATTGGCTGGTGAACTCAGCAGGAACTTTACGTTCTATACCTCACGGCGTTGTGACTCAAATGTTCCGAAGGTGAATGCGCGCAGTGTGTCTATAATACGTCTGTCAGGCGCCAAGCCCGCCATTTTCACTGTAGAGTCCCACGAGCAAACGTCTAAAAAATTGGGATTTTTTATCTGCGCAAGTTTGTTGACTAGCCTTATTGTGAACACGACCAGGTTTCACACA

>Of_kirrelL_CRE

ATATATAGATAGATATATATATAGATAATTATATATTTTTAAAAAGCTTGTTTTGTTTACTTGTTTTAAAGTTAAAAGTAATGACCAATGCGCTTCTCTGGGCATGTTTTATTACATGAATACTACACGGTACTTACGTTGTTATCTGTGATTGAACATGTCAAGGGAAGAGAGATAACGACATATACTAGGTCCGATAATTAATATAGTTAATTAACAAACAGACAAAGTTGAAATAAGATATGATTACTGATCAAACGAATAATTAATCACAATGATACAGTTACTGAAATGGTTCAGGATTCCTTCCCAAGGTCTTCGATGTTATTATGCAAATTCACGCAATTAATGGGCACACGCCTAAAGTAAGTTTGATTCGTCATTAGATACTAATGGGTTTTCCTATAATAGCTGAATTCAATGCAGCTGTTATGCTATATACCTTGCAGTGACTTTCATTCGAATAATGGTTGTGTAATTAAAATTGTCAAAATCAAGGCCTTTGTTTCCTTCTAACATCTTTATTGTTTTAAGACTGGTATCTAAGATCTAGAAGAAATGGAACGTCTAAGACCTATCTCATTTAATAAACATTCTCGAAATTACTACCAAACAACTCAGTGCTTTTTGGCACAGACGTTTGTTTTGGAAACAATCCAAACGTATTTCAAGAAGTAAACAAGTACTTCGTCGAACTCTCGAACTTGTTTGTGGGGGATCATTTCCCATCAACCTTTGCGAAAGAACAGGGGGCTCATGACCAGTCGAGGAAGTCGGCTAATTTAATTATACTGGTCATACGAGCGCTCTGCAGTAATTCATATATTTCACTACTACCGAAGGGGTAAGCGTGCAGATGAACAAGTGCTAGCTGACAGTGTGAGACAGGACTGAGTGAGATCCGGCTGCAAAGGATTGACTATCTCCTCACTCGGTTTACTGTCAAGTAGGGAGACAGACTATACTTACAACTGAAGAGCAGTTGGAGTAAGACTTCTCTTCTAAGGCATTCATATCAAATACAGCGTTTACGTGAACTTTTGCAACTGAACATTGCTTAGAATAAAA

>Aj_kirrelL_CRE

aaagcatcactgtaagacagtttggagtttaactctttatgtgtaacaccgctctagaacttataataaagtgaataattattttccaatatatcacaagtagaaaaataacacgctagccagactattttccaacttttaacaaaatccggttcgtgtagtgcaccttgggtaatcaatcattcaatagaaataatataactaattcttccgtaagaaaaaaatgtttggcagagacatcaatacttcctttttcatgccaagaaacgatatggttaggcctaatatttattcaccgatgtagtttagaatccagaaatcatgaaaagaattcattttctggactgtacaaatgtataataatttaaattttgttcataataaaagtacagtatcgaaataagaaaaacttaaaactgctatactcgaaactgtcaacaacgtgagtgagatttattggttttgttgtaatgaaatatttaaatatatccacaaaatgctgtagttttcatagatcgagaaagcttggggtgtgttggatataaacagtcacatgacttggttagagcgatataattgaattagaattgatgaaatgctaagacagcaaaatcatacgtaaaggaagcataaattaagccaagttgttgtgtgtgccaatgaacacaacgtatgagtaatgcatagtagtgaattttcatcggcatcagaacacatgaaattttataagttttaaagtagaagcattttgtttttgagattttaaaaacaaaattatcagtttacaattcttacatacaccgaaggttaatctctatcgaataaacgccgaaaaaaaggactagtactgaagacgttttgaggcttacaacttctaatttcgagggagagcgtc
