# Supplementary material for: Architecture and evolution of the cis-regulatory system of the echinoderm kirrelL gene
Source: eLife. 2022 Feb 25;11:e72834. doi: 10.7554/eLife.72834 (PMC8903837; doi:10.7554/eLife.72834)
Supplement: Figure 6—source data 3. [file elife-72834-fig6-data3.docx]

**Figure 6 – source data 3**: Echinoderm PMC-specific Ig-domain protein sequences from used for tree construction. *Strongylocentrotus purpuratus* (Sp), *Lytechinus variegatus* (Lv), *Eucidaris tribuloides (Et), Parastichopus parvimensis (Pp), Patiria miniata (Pm), Acanthaster planci (Aplc), Ophionereis fasciata (Of), and Anneissia japonica (Aj)*

>Sp_KirrelL

MNHICWIPVLLCAVVLTRYARAGFIVEPFSTEGIQSETVVLPCTILNEPGAIVVWYRNKAQLSIDKTLVESLDSDVQARFSVVGNQTFGEFNLQITNVKPADESQYYCSFISRFGGASSVVATLTVLIPPSEGYPRCSVTAETHRILRMPSGQVKWLLLTCESLGGDPAANLTWYRKNWTVSHATKHTNVYRRILTPGDNGVTFICEATSSALREPRRCEVTPMSIPPEVHVRPLTNSVPFGTNATFHCEVSAITRVQEYNWFIEGTLLTPETPGIVFGNNGKVLVIANVQMEHDSKPVECEAVIKNRLVARASTTVVVIPPRKTSPPLPSNPTEKVTIIQVEKSEVPTSHPILSANMNLIIVIVSGSLIVLLLLIAIIMGCWVFGFRKPRRPHTRVNIDYGEVPVDMPLDGISHYSVPAALFSTEIPDSRLNESFHQPRHYDENTSGYISALYATLDKNKRYPPKMTSSTSLPEKPVAQITPTAGRNVVPHFAAASTIDKRKSTGAIAYSDLKDLDNDPDFKKGCSCDCKADDSGKEDPETEQ

>Lv_KirrelL

MNHICCIPVLLCAVVWTHYARAGFIVEPSSIEGIQSETVVLPCTIRNEPGAIVVWYRNKAQLSIDNNLVNSLDPDVRARFSVVGNGTSGEFNLQIVNVKPADESQYYCSFISRSGGASSAVATLTVLIPPSEGYPKCSVTAETPQNTKNAFWPGEVALLSCESMGGDPAANLTWYRKNWTMSHAAKHTNIYRRILTPGDNGVTFICEATSSALREPRRCEVTPMSIPPEVHVRPLTNSVQFGTNATFHCEVSAITRVQEYNWFIEGNLLTPETPGVVFGNNGKVLVIANVQMEHDSKPVECEAVIKNRLVARAGTTVVVIPPRKTAPPIPSRPTEKVTIIQVEKSEVPTSHPILSANMNLIIVIVSGSLIVLLLLIAIIMGCWVFGFRKPRRPNTRVNIDYGEVPAEMPLDGISHYSVPAALFSTEIPDSRLNESFHQPRHFDENASGYISALYATLDKNKRYPPKTNSASSLPEKPVAQITPTAGRVVPHFAAASTIDKRKSTGAIAYSDLKDLDNDPDFKKGCSCDCKSADYDDNEDPATEQ

>Et_KirrelL

MKVQALFYAIWAAVLTGSVRAAFMVQPESTAGIKSETVTLKCQITNESGAIVVWYKNKAQLSIDRNLVDSLDHDVRSRYSVVGNRNEGEFNLQILNVKPSDEGQYYCSYISRAGGGTSAIGTITVLIPPSDGYPRCTVYAETPQNGETSFWPGEKASLSCQSAGGDPSANLTWYRKNWTVSEPTKRRNVYRRILSPGDNGITFVCEATSPALREPRRCTVTPMSIPPEVHVRPLSASVLVGDNVTFHCEVSAITRVQEYNWYIKGTLVSAETNGINFTSNGKMMVISDVQMNLNRKPVECEAVIENDLRSRARTNVEVIPPKKTPPPASTPTQKVTVGEVEVDETEIPTSSGGPLFTADMNLIIVVVSGSLIVLLLLIAIIMGCWVFGFRQPRRPNTRVNIDYGEVPADMSLDGISHYSMAAMFSTELPESHNNDVIRNPGNIDENAPGFISALYATLDKNKKYPVKMTSASSLPEKPIAQITPTSSKAAISHFASASGLEKRKSTGGAIALSDLKDSVDDPSFVKGCSCKDELEKEDPNTEQ

>Pm_KirrelL

MTMWSPRAWFVSAVAVIVLSQETQAIFTVLPANISAIAGDTVTFRCTALSSLDTFLYWSKGNTHLSLGGKLLHSLDNDLRERYSLGGNQSIGEYDLRIENVKKTDAGDYQCSYIGSAGGRASPPATMTVYVPPLAKYPLCKVRTQSQTSETTGTMWPGDGAEFTCQSSGGDPPALLQWQRINNTILEPQPSHIVYARILLPEDNGVRFTCVATGPALREPRRCSVMPMRILPVVQVRPLTRMIIPGDNATFHCEGSAIPHVASYSWQVAGNHISDSQAGFSFANQKQLLTIHDVKQSHDQIEVACEASTPTGIAESASVKMVVTPIYVPPTLPSEGTPIDFIPSAPAGPDDTSVDEEEPGIIDGDLFMITLTIFGALVILLLVVAVVMGCWALHAKGKRPQTLKRPRVEYQESTATTIDLEPLPLPTQLAYEPPTTVSSDPMAIPAPPYTADKENAPGYISQLYSTLDSSRLPARPAFSGFPEKPVASITPTQGVASTLPRPGKIDKPCGTGKPTVKECPASFSIPEEKVTSQEDKMNEENEDRKNGN

>Aplc_KirrelL

MTVTMSLPELQLIFAVVAIVLTPKTEAMFTVLPANISAIAGDTVTFRCTAHSTLDTLQYWSKGNTHLSFGGDLADSLDNDTRSRYSLSGNQSVGEYDLTIANVKKEDGGHYRCSYIGLAGGKSSPPATMTVYVPPPARYPLCNVRAESKSSKAASTLWPGDGAEFTCQSSGGDPPALLQWLRTNRTILEPQPSNIVYPRILLPEDNGVRFTCVATSPALREPRRCSVMPMRILPVVQVRPLTRMIIPGDNATFHCEGSAIPHVASYSWRVAGKHISEGQAGISFANQRQLLTIHDVKQAHNQIEVSCEASTSTGIAESASVQMVVTPIYVPPTVSTEGIRIDFIPSVPALPDGPEVKEEVPSILKSDLFLIILTVFGALVVLLLVVAVVMGCWALNAKGKRPQTQRRPRVEYQESTTTALDMEPLPLPTQLAYEPPTTVSSDPMAIPTTPSTEDREHAPGYLSQLLDSTRLPARTAFGGSLDKPVASVTPTPGVASTLPRTGKVDKPCGMTKPTIKECPASFSIPEEKEKGYKVSDGNDKNNGH

>Pp_KirrelL

MSEVYLILLSVTVTQLFTSCLGNFLIQPEDSTSVVGEVVIFKCSVNRTDLDSTLLYWSKDKHHLSVNERLVESLDRGVKSRLSVVGNRTLGEYNLQIENIRRTDVGSYVCSYIGLSGGQTSRVASLTVLIPPRKEFPLCFILAQTLRTTGQVDTFWPGQTARLRCVSVGGEPAATLVWKRKNATISSTKTSESIHERVLTPSDNGVAFTCEASSPALKVPKKCEVVPLKLLPSVQIRLMTREVIEGSNATYHCEASAIPKISLISWSFAGREVGFNESERVVVSEDKQFITIIDIRADEDQTSIDCVVSISTGLQSRDTMKMLVTRKPIIATNHPATASPDDRLQNADNFPWNSQLHVIVIMVAGSCVGVLIFVFSILICWGFSRAKKPRRPNTRLQVDHVNPSSNELGLEPLTYPGQIVYNTLPDCDTLPRRGGDSFRNSLHDENSTGYLSSLYASIDRRGGYTPRFGSLRGDEKPVAQITPTTSLKRPIIPYSAQPLIEQDKDSYKDSSSRDSVSSAGETEIF

>Aj_kirrelL

MDMLTKLLCLAVLPLLAQAKFHQTPQSQVVILGETVTLRCAVTYIPELVIYWKKGPAHISIGRNILADIGVENIRRYSIVGDELSGEYFLKIVDVKVEDAGDYKCAYVSKTSGATSLPAKVDIIIPPKADFPVCLSTPDPGAHKPGSTVELNCISLGGIPRANLTWTREGVILGNVTSNNNKVTRVLTPKDNGVYFTCSATSPALRKHRKCTIMPMKIPPSPMVKVSANVSLVGDNITASCHSEGIPAIGSYTWFIDGRPIDFVIPRFSAINHHLKIRNLRMNDNNAILTCMVTTTLGLTGNYSTLLKVELPTTTVLPTTSMTPTKNTDTATELFSIIDDENNAGTGVNTKNDIDTSVTRYNMKLILALIFGMLIFIFIIVLMTLTYFFASHTTRPRQKKSSYEDSTLGPVSHRNSFIYMPALHQGENGQIFQQSLANDENSSGYISQLYASLEKPKHHMSKSQSVSALHNKHMKSVLSPSPSVVTLPVNTPCGGRKDFRNFNKVHYTKQPLRYTTHVDAPFTIPDDGMVTFHPLRRNTMDNFASTSNVANRGAYAIRRGSGYENVRLHDNFGHAEMNYAPNEDIYVNRSIDEYRNNEISDDFPPPPPYVAEDQSEPNPFLPD

>Sp_Kirrel2L MACCWNLYIVILTLMMMQWKLILGGIFKHGPSDTVLVQGKTAVLRCAVKTQGNEVVYWFHVDSGQYISYDENIYSFVMEDRYSIIGNHKKGEYYLTIRSARKSDKGEYRCVYGNQFISARMVVLVPPNDGSPACEVLPAADTTSIGNVAQLSCTSQGGDPPAELSWYREDKLIAGPTVHANSVQRLVKQEDNGVLFTCSATTPALTYPRTCTVMVLKNPPMVTVTPPEVNIKHGETAQFTCRGTGETNVTGYRWFINYVVVGSKVSSPRYTLDPTNTTLTIHNVQAWENGVIVSCEANIQSGLKGRGSANLAVILPPNFKTASPPISNNGNKGNGTRLSPKGLHPQPFMTTNIAAIAGASAGALVLLIVVSTLAACCAKGKHKYPAGKNIPGSLRYKTSQQILIEEDFPIYAKPMKVKRLNCGVKEPSRPNSNSTTAESALTGVILDVGQGVAIPLCLRPPSLKRLGRKITSNYEKIGFDGPLPLTRPEPMGSEDKDSVPVEGAARPTEICRSQNNATNGDLRNGTSKPGELVYADLDLKENNNDSSAKQTQSDGVAYAKLRL

>Lv_Kirrel2L MASCWNLWFVAVILMVNQWKLSLGGIFRHGPSDTVLVQGKTAVLRCAVKTQGSEVVYWFHVDSGQYISYDENIYSFVMEDRYSIIGNHKKGEYYLTIRNARKSDKGEYRCVYGNQFASARMVVLVPPNDGSPACEALPAADSTSIGNVAQLSCTSQGGDPPAELSWYRQDKLIAGPAVHANSIQRLVKTEDNGVIFTCSATTPALTYPRTCSVMILKNPPMVTVTPPEVNIKHGQTAQFSCQGIAESNVTGYRWFVNYVVVGSKVSSPRYTLDPTNTTLTIHNVQAWENGVIVSCEANIQSGLKGRGSANLGVKLPPNFRTASPPISNNGNNSNGTGLSPKGLHPQPFITTNIAAIAGASAGALVLLIVVSTLAACCAKGKSHKYPAGKNMPGSLRYKTSQQVLIEEDFPIYAKPMKVKRHNCEVTESSRPNSISKASTAESALTGVGSDAGHGVAIPLCLRPPSLKRLGRKITSNYEKIGFDGPLPLTRPEPMGSEDKDSAPVENPLRQTELCRSQNNATNGELRNGTSKPGELVYADLDLAENNNDSSTKQTQPNSKASDAVAYAKLRL

>Et_Kirrel2L

MNLLLPLICFSVFTLNTCHARRPTFRYGPTDTVLVEGKTAVLRCAIRTQANEVVYWFHVDTGQYLSYDHNIYSFVMEDRYSIIGNHKKAGEYFLTIRNTRKSDKGEYRCVYGNQFRSARLVVLVPPDGGSPACEVMPAADSTSPGNVAQLTCTAHGGDPPAELSWYRNDKKIAGPSVHSNSLQRLVTEDDNGVLFTCSASTPALTYPRTCSVMVLNKPPVAKITPKDVTVKHGRTATFTCSGTGVPSVTLYRWFVNYVIVGSKVSSPRYNLDLTNTTLTINNVQSWENGVVVTCEVSIASGLKGRASANLVVQLPPNFRTNPPPVPDWNQGGGNNTKLTPRGLEPRPVMTPNMAAIAGASAGALALLIIVILLAVYCARGKSTKYQPNKNIPGPQLRYRKSQQMLIEDDFPIYAKPIKIKRSNIEVKDSSRPSSDSTTASTAESALTGESSVRSDHVNVVAIPLCLRPPSLKRFGRKTTSNYEKIGLDGVLPLTRPEPMGSESKDSVTRESSTRQNPLGTKVSATSDTGDKPNVEGLVYADLDLVENNNETSSKQTQTSLKHTDGVAYAKLRL

>Of_kirrel2L

MALSQLFQIGIFFQAIFLSCALIFRYGPTDTTMAVGKTAVLQCAVVLQSNNEKVSWFHVESNQILSHDENILTLEDRYSIIGNREGKGEYYLTIRNVQRFDEGEYRCISGSDFRSATLKVIVPPPQDYPVCDVSPAPAGNQPGLLTQLTCTSDGGDPPARLTWKRHGIIISPTVSHANQLQRVVYEEDNGVPYTCFASSAALEYPRSCTVMVLNIPPRAEIKPMEQEVRAGDTATFECFGYGIPEVTSYQWLVNYAPVSEERNPRYSVDKTNRTLRISNVRRWEHGSLIICEVSIESGLKGRANARLLVHSPPVTVAKGSGPIVTLAPTAHPNMHPNPEQPKGRFHITGHAGALIGSIIGTLLLLTIVLVLTCLVLRAKLSQMGVDIIPFCKEKDKSPNGDNFPIYAKPNKIRNDAPPLPDAPRPDRSTLTRMATLPRAADDLEKKPKRKIKKHFSNYERIDLVPRQVSFLETKNDLDQETDLTLVTSTPNAEGIVYADLELNKGGIPAPDVVSSNRPDMVAYAQLRL

>Pm_Kirrel2L

MSCRKRGLVVLALRTLLFSLSSAAVVFRDSPEDLVLAAGNTAVLRCSIQAGGGERDTVKWMHLESDQVLSKNEAGVRPASGEYRITSNRRGGEYHLRIIGVQLTDRGEYRCMCGSEYRSAILTVVVPPDTGYPLCHVQPNPTQQQPGRIAELTCNSHGGTPPAALIWHRKGEPISEVTTHSNALKSLVTAEDNGVVFTCFAQSKAASLPVRRCEVMVLDTPPTGSINPDLHVVYPGDSASYTCTGAGIPKVIRYRWLVNSIDVTRAENRGQRYEVLDNSRTLRITDVRSWEDGAVIACEVSIASGLTGRARAKLSIALQPVTSAANEANPGALFPINDRVPTENDKKTGPTGLKPQGNTATAGVVAGATMATVGIILALVAIAILLLKIKASGHFSMANANEKKCFPDKLDLSTGTIKKVDECPIYAKPNKLRKGPAPAPPGAAAAVLAACDLAMQKKQLRKNHHSYEKVEILGCSSPGQTYTLPVRLQARAPTTPKTRRSDTLRCIKAQAPLPPNLPPLPRSPLPPLPQKSPLPTPTPSDWPTGDLVYADLDLPAKTETVNQSHPSFVGVDYAMLQGRNSGSSGGYDWGTS

>Aplc_Kirrel2L

MSARRVATPVVLQTILAAVSAAVVFLDSPEDLVLAAGNTAVLRCSVEPQGTEREPVKWVHLESNRILSEDSVVMGPTRDKYSVVRNHGGGEYTLKISAIQLGDEGEYRCMCGSEYRSAVLTVVVPPDAGYPLCHVQPNPTKQRPGGTAELSCTSHGGTPPADLVWHRGDLLISDVTTQANALQHMVTASDNGVRFTCFAKSKAPSLPVRKCETMVLNIPPEASISPQLHVVQPGESVTYTCAGAGIPAVTGYRWLVNYIDVTGEENRGQRYKLLDDNRTLRITDARSWEDGALIACEVSTHSGLIGRAWAKLSINSPPVMSTTKEVNPGLLLPTTDNVPIENDKKKIGPTVLKPQTNSATAGVVAGAILATVGIILALVVIAILLTKTKASGQFSMATANKKKCFEDKPGLSTGTIKKMDECPIYAKPDKLRKGPAPVPPSAAAPAPECALATQKKKARKNHHSYEKVDILGCSLPGQAYTLPVRLQSKAPATPTTRRSDTSRLLKAQAPPPPGFPLPPLPRKSPVPTPQHPPNNELLYADLDLPATRETINQSHNSTAGVDYAILPVHNSVNSGGYDWGTS

>Sp_Hypp1164

MAWLSVTILLVTLMFSGALGLSFRNQPDTVTVYAGDTIYMRCDLNLDGVAGSSVSVTWIKSDNTNPQTQYISIRGHVVHTYTRFSIAGDSNLGEHDLMIRNVDYEDAGMYWCQASGAGGVIESVAGRLTVRVPIPIPTCSMTPPQPSVGQRVTVICSQQMHPSVNYEALNWWNETSRSKIRTQGRYSDGSIFFTRLLSDTDQGQQFVCSQSSVFVEGERNCTVVPLPAIEVSTIANPLETIVMNPTVKYVTEGSSAMFGCQSRAGPVARWVFGYGSRASKIRQTRGRFLVSADRSEFTVIRTALNDNGTAVRCIVILPSGVKLIGESTLKVLPADTQPAAVNPRKPPPPPPVSTRQTFRTEQPPIIPTRKPSTDQEPSTDGTSHGASNAANTETDSHPNLSGGKTIVNGQTTTKMPTATSKTTTKAAETSTRRDVVQIGANTDIDSGVIATEANVNLNHAGAPKIQTGGGYRQGGGNTHQVSNKDLPDQESNGSGIAGAIIGSLLIIALIVLIIFLIKTKRYPKSVPTWIPPHVVAALKRPIDMVKKSGSAGGQSGSSVKSSKRNAQMRMKEIEVVVNPPPPEWQFRLKDVESNSGGQVGGYALTPRQSHMMDGPVYANITPDLSKAPPGQDEHDLDDDDLDDDSVFSSDFDDDLDLEGEIAAELVARSCESEGNTDEFIEERKSKNVEGLVYAQLDMGPNAVKRSTVFIEGEKTQYAHIKVGNSAKAK

>Lv_Hypp1164

MERTILLFTLMFSAALGLSFRNQPDTVTAYAGDTIYMRCDLNLGEVAGSSVHVTWIKSDNKNPTTYISIRGHFVHSFIGRFSIAGDTTRGEHDLMIQDVQYEDAGLYYCQVSGPGGTVITSVMGRLTVRVPIPIPTCSMTPSSPRVGQRVTFICRQQMHPSVDYEVLNWWNETSRSRIQQQGRYSDGSIFFTRLLSDSDQGQQFVCSQSQVFVEGERNCTVVPLPAAEVSTIANPLETIVINPTVKYVAEGSSATFGCQSRAAPVSRWVFGYGSKASKVRQSRGRFLVSADRSEFTVIRTTSNDNHTAVRCIVLLPSGEKLVAESTLKVLPSDTQPSAGNSRLPPPPPIITTQATTTERPSTRPTRMTSNGPTGHRDTPTVNDPSIGQGTSNKANTGLGLQPDFSDGKSVSNVQTTPTKAEVTTKVTSKPAETSTRRDVVQIGANTDIDPGVLATDANVNQNRAEAPKIQTGGGYRVIDGGNTHQVSNKERPDQGSSSSGIVGAVMGSLLIIALIVLIIFLIKTKRYPKSVPTWIPPHVVAALKRPIDMVKKSSSDAGQGGGSEKSFKGNAPMRMKQIEVVVNPPPPEWQFRLKDVESNSGQVGTYAVTPRESHMLDGPVYANITPDLSKTQTHAVQDDHELDDDDDDSLFSSDFDDDLDLEGEIAAELVARSCESGGNTEDSIEQKKSKNVEGLVYAQLDLGPDAEKRSTVFIEGEKTQYAHIKVGNPVKPRSQRH

>Et_Hypp1164

MAPLRAVDVIFTVFISAILITCALGLRFRNEPGTVTVYAGETVYFRCDVYTNQGAPPANLRVTWMKADSRNARKIISVSGVIVSAFADPNRFSQVGDPELGEHDLMITNVQYEDAGMYWCQVSTSGQYLESRMGRLTVQVPVPIPVCSVDQPTPTVGQRVTFNCQQQMHPEVNYEALNWWDETTRRKVVTQRRYSDGSISFVRVLTESDRNRKFVCIQGLSFVEGQRNCSVLPLPAPQISTPANLAQTIIMNPEVKYVTEGSSATFGCTSSAGPVSRWIFGYGSSSTKIRQTYDRFLVSSDRSELTIIRTTLRDNGRAVRCVVLTSTGDKLIGQSVLNVLAPGTEPAATIPKIPPPPPSTSRPTTPTKPPTTKAETTQNDTPKQSTSHTQNTETVSQTTTTNTGNKGNTETNVNATTNQEPSTRRDAGQIGASNPDVITDAETKTPPNTEQNNANAPKIQSGGVYRSSNPHQVSSDNNGNQRDSSAAIVGAVLGSLLLIALVVLAVFLVKTKRYPKTMPVWFPPALLAVLKRPSKKQDGATGNENGPEVITSGKQSIRLKRIEVVVNPPPPDWQFRLKDVEGGDGKSVAPLNNYETHEGPVYANLTPELTKACPEEDPDDDDNDSLFSSDFDDDMDLEGEISATLAQMAANPEPIYENRMSLGPLPVMTSVPATAPVPAPAPSPNERKSKNVEGLVYAQLDLGPDAVKRSTIFIEGEKTQYAHIQHGNSAKRK

>Aplc_Hypp1164

MVWYGGVVFVTAFLSLAAAVNFLSGPSNTVVPVGGTARLKCVADLRTVRGTVTLYWYKAQDGGGYYITGGSDIYSRNLGAERYSLETSSVNGEYSYTLKITSVEAVDSGQYQCMGVDSSGHTRSPRGSLSVVQAATDEPLTCSFYPQSPTVGQTVTFSCIAPRGIAPEMLSWWMAHSVRIQPSAHLSNNHGISFVKTLSNADNFAEFTCLVGSSFDTTNNMNCSVTPLAVPIRAEVTAETAFVVADRTATFRCRGTAIPPVTRYRWIYGSGRNTIKISTSRGRYTVINNGDSSTLTIAGITMQDNGQTVRCVVLNEVTKTIGSSTLRVTNRIQTTQKTTILTSSSTSPMYSTVLTETPTASLDPSQPTTSVRVTGDILVSTNQQIHTTNDHGDVINIVGCKDCGGKISPQQNPGDEEKSSNSTLAVALGILAICALVAGIIAYIVYTKRGRTSGRVIKRKWKNKPLQRLRSLRQSVSSSNQDVVNPNSVRLKTIEVIVNQPPPEWQLRVKDVAGEEKEPREQDLPKLGNDQSPVIQVTPDCALPENCDALYAKPDKMKKKRRHHGDEDGNDPRPVTASPTVPGQYLTADADDCFSDGVSDASTWDMSSVDSGCEEEMDEIRQDATAYAELDLTVAAERSKECTPPTETVEYANTTVTKTSLPRS

>Pm_Hypp1164

MLCYGIVLVTIALSSVTALDFVSGPGSVIIPAGGTARLKCVADLRTVRGSLTLYWYKAQDGGGYYITGGSEVYARNLGAERYSLETSSVNGEYSYILKIAAVEAVDSGQYRCMGVDAGGSTSSPRGTLSVVQAATSEPLTCSYYPESPSLGQTVTFSCTAPRGISPEMLSWWMAGTVRIQPSANPSNNHGISFVKTLDNNDNFAEFTCLVGTSFDTANNMNCSVMPLAVPIRADVTPQTALALLGGSATFRCRGTAIPRVTRYRWIYGSGVSTAKIATSRGRFTVINNGDSSTLTIGGISMQDNDLSVRCVVMNEITKTIGSATIQVTTHIQTTRATTSLMSPSISPVGTTEKPTESLNPIQPREPMDPSQPTPSIRTTGKVLGSTNQQISTTNDNGDVINILGCGKECGEKLSPQQSPDGEEGSSNSALAAALSVLAICALATGVIVYIAYTKRGRTSGRVTKRKRKNKPLKRLRSLRLSVSNSKPELINPDSVRLKTIEVIVNSPPPEWQHRVRDVESETKEVQEQDLPRLEVTPDCDALYAKPDKTRKKRHHGDAVGDDTHPVATAPPAVPGQYLSADADDCLSDDVSDASTWDMSSVDSGCEEETDEIREDAAAYTELDLTKAGERDNKCIPLTEAIEYENTIITKASLPRN

>Of_hypp1164

MEWSAGLFRLHRHLRVLCLTLVGIYYLVNLVESLSISEQPRNRGVQEGGTFTLNCVVESTNQAYVIWYKVESPGSSVPLSTYRTIRPEAPDSERLSIVGSGNVYNLKVINARNADESSYQCVAYSRGSSVRSEVAKVFILSVGDSVDCHLYPQHVRVGANLNYTCRAPESLPTATLTWWRGDQPMSAVETLPDGALQLRYVISEYDNFVRFTCTVGVDFTQAYGLNCSSVPLHVPLDGNLNPHLNRVSVGSNAAFRCSAVAAPPVNKIKWLVGYGSNSKISGTGGRYEVKGEPRDHTASTLIIKNIAQADNGTTVRCVVMNEIRQKDIAMAYVIVSDTNDPKVAPPPTVSPAIRTTRRPSRTNHLPTTKRTTTELTTKILTRLYQPGLKDVEISVHDGIPTRDIIPSVNNDGISEGNTENSSPSSNSNLAPTVAAICVLLVLLIAGGGIAVYFLIFRRKIVNKNKFNGEVKYRQSSDPNGGELTAEERASARMSLRLQTIEVVVNQPPPDWQCKLQDVKPNENEYTHFTSKANEIHGSPPPAYTKTPTDTERRSSRDEQDGGDKQNGSDEEKENDAIYALPAKKPSTGKRDGDTHQPAKRQISESKKRLLSQDYNDNENEEDEDIEWDQSSWDNSSFEDETETVERIQEGRMYSGPGEKIYANATEEGALNDYGPEYGNLPNLNVDETDPTYSRVASLKE

>Sp_IgTM

MMILVSCWLMLAMSKMAYAVDLLMTPPVVQVYQAQTFRVTCVVTGEYQARYHSIHILHLPPKGKETYVTNDETIARTLNQNRQLRYSIFKKTSGSGANQRWEFMLTISKATKADVGRYQCLRIERAPRTTQYSTTFSRVFVNDIIAASQVAPQCALSPANPSPGQTTTFSCFSIGEAPKLTLTWTKGKKALPSKSSNSSGKFNVVTFQHIISEWDNNATFTCTATGLALGYETRTCSVVPFVIPTNVTVTSTQRVKSGKPAEFICLAVSVPATNKYTWVITKGSGKEIVDRSSGRYVIEQGGALLRINSVTPSDNNSSVHCIASNGMDIEGTSTGTLFTEPAKPSTGPGGGGIGSRHGKGDGNFAFFGKGGGSGLTPHPGLFTPGNGRRDIGTNGFGGERPNVPLLVGGTAGFVIFMTLIVLLLAMVMRTRTKRRKTITKLDVQRRPISIISLSNAFDVEKADVALVEANTTTQEKAVPRSTSLRKSVHSVSSRKSSINQDISRNQLADKLAATLRSKSYVSLVETPIPTQPGQSDKLDDDDCVSRVSTEDSAYDELTCSVSSSVVLSSRSNSNTASQSCYGSLGSLPSSVRYSRGKDALEVFKNPNLHYAEIDWSGRMPPSGKIVDGEEKSLYAQIMKK

>Lv_IgTM

MRVLVSCWIMLAMSRMACAVDLLMTPPLVQVYQGQTFRVTCVVTGEYQARYHSIHLLHHPRNGRETYVTNDETVARTLDPNRQIRYSIHKKTSGSGQNQRWEFILTVVKAAKADIGRFQCLRIERAPRSTQHSSTFTRVYVNDVVPASPVAPQCALSPANPLPGQRTTFSCFSIGEAPKLTLNWSRGKRRLPSKSSNSTGKFNVVTYQHVISEWDNNATFTCTATGLALSYETRTCSVVPFVINTNVTVTSTQRVKEKEPAEFICLAVSVPATSKYTWVITQGSKKEVVDRSSGRYVIEQGGALLRINSVSLEDNNSSVECIASNGMNMEGTSTGTLFTEPATTSPRPRGDGIGTRHGHGEKGDRNFVFYSNGGDPGRTPHPSSPENEIEKNGFGGRKPYIPLFVGGTTGFVIFVTLIVLLLAMVMRTRSKRQKAVITSDAQRRPISIISLTTAFDVEKPDVALVEAKTTPQSQTVLKSTAYRKSLHNLPSSSSRKSGANQDISRQHLADKLSASLRSKSYISLMETPIPTQHTLHSDDATDDDCVSRVSTEDSAYDDLTCSLGSVSALGSRSNSNATSNSQSCYGSVGSLLSTVRYSRGNDTLEVMKKRSLHYAEIDWSGRTPPSGKIVDSDEKTLYAQIRK

>Et_IgTM

MKGDLLGFKMSLMLYLPVGIILFVGRLTLAVDLLMIPPIVHVYETQPFVVTCVVTGEYQAILHTIHIVHHAANGKETIITQDETIHSIVDAGRAQRYGVTKKTFGSGADQRWEFTLAVSRASKVDVGRYQCLRTEESSQKRQLSPTFSHVHVNVVVPAGPNVPRCSIQPAEPLPGQTATFSCYSFGEAPKLTLTWKRGPEKFSTQANSTGKFNVISFTRSLSPWDNNATFTCTARGIAIGSETRNCTIVPFKIKTNVTVTSVQKVRHGEVAEFLCLAKAIPPATKYTWIVSKGTHEVTINRSHGKYEVEQSGALLKITAAASDNNSTVRCVAHNGIGIDGMSSGTLFTEPSTSPSGPNRGGGRGGIFNKHGNGDSVFFDKDGADGTNNGSSGTSDGGSNIQNPGKNDIGSNITSGHHPNVALLVGGAFGFISFMALVVFLLAVVMRSRGRRQKHVPAIRRQSSVRSIIADNRQNAEIAMIRETSAPHEKLRGSAATYRSTLQPQGVRKSALYSEVSRTQLADKLAATLRTKSYVALLDSPLPSRPGEENLTNTDDDDCVSRESTESTFADSAYDELSCSMTSTVLTSRSNSNSSPRPKDNSSNESASLPSSIRYSRGKDQLVGTSATNSKLLYAEIDWSDRKPPSGQILSGDEKTLYAKIRKK

>Aplc_IgTM

MPPQMVTVGDSAEFFCLATSVPPSNRYRWFVGRGQSMTRVSKTKGRFVIANQGASLRILNITREDSGMPVRCVARNPLDMKGMDEDMLQVLIQRDPIPTTTIAPTKPPRTTTKSPPTPSPTTLPLTVLLKTSQRIRLQTLRAQATKAPSLAPPEQPTLRTASKQQPLTQSPEPKNPVTTVLVTTNKMDEPVSASPPLQDVTIPHVPPDSVLQPGPKGKGFTAGAAVGFTVLVLIVLALTGILIKIKFVDKHEKPVKRKTLRPANKPRIDKLDIVVVPNATPEEDGNTNNAQPSNNTDATPKPARSARRQSFVHPDFLEQLASTIRSKSHCSSMFKRGSFSWRKRKASTLDRPHTVKSEDPALDTEATSTVKRYSAFFNAVPQLSSDEMPVATARQVQKTESVYENTEIGLRLQQQVNRRSRDRESPYENTQCPMRDAAPLSDPDRRKSEASSLLDLVYADLDWTGFSSNQDEVNDTEEKTEYAAIRTSKVLS

>Pm_IgTM

MESRLIFTSLVLTAVYAAELRVKPSDSTVTIGQPHTFECIIAGEYQKGKQHVYWAWTGPRNQQKFLSVDRTLYPAVDADKRSRYSITGNMNEGEFNLHISSTTADDQGQFRCIMYDSGRSARASAMLSVQEPRLPHQGYPKCSITPTNPQPGEYATFSCISAGGNPPATLSWARRKKSDIPAHHNSTGQQTGAYLHRLLTGHDNNVTFTCFSRSAHSADFGSCTVVPFEIPTHVTIMPPQMVAVGDFAEFFCLATSVPSSIKYRWFVGERQSMTKVTRTKGRFVLANEGASLRILNITQDDNGMPVQCVARNPLDIKGMDADIVRVVVQRNPIPTVTTTKPLLRSTKATPTTSTPFVPPRTSRKTWMQTFTVQATEFQTPAPPKHPPLRTAPKRVPESPKPKNSAAIIPVTTNEVDEPVSPSPPLEDDTTRQPPDSVQQPGPNSKGFTAGAAVGFTMLILIVFVLIGILIKIKIVDKQEKPAERKTLRPAYKPRIDKLDIVVLPNTAQENDSNTSDVQRSSSDDAPRSKHKQNSVKSDKSPRRSSFVHPDFLEQLASTIRSKSHCSSMFKRGSISWRKRKASTLDRPSAVKSESSLAEEATSTVKRYSAFFNAVPKVPNEEVPVASPRNIQTPESVYENTEIGLKLQEEANRFSRESPYENTQYPMNDTALSSDPDNRKSEASSLLNLVYADLDWTGFPSRHEDVNDTEERTEYAAIRTSKVL

>Of_IgTM

MEQLATWKYLSFCFILTLISGVIEAVEIVLHPSSASGVQGSTITLRCGVNTEFRAGVHRIYWAFYGTPGQHTFLSVDRSIYSSLTSSKRNRYSIGGGVGLGQIEFNLQISNAQLEDNGVYECILYDPFTGQRVTTPDPARVVVLPLRPPDRGSPECRLSPPNPRPGVIVTFTCITSEGLPRPTLTWRKQGTILPGVLNSTDDLGLEILQFKRYISNWDNNATFTCTAASPAMPESRNCTVKPFEVPIHVSVTSLQLVKVGQSAEFYCTASGVPDVNRYRWFIGRGDTAERITRTQGRYTLSTQGTYLRINDVTEKDNGLPVRCVARNAIDLRGVDEGVLRVIVPRPTVNVVNNPTTVGILTTPLPKPVTQSRNGHDFKPAVGTNPVGTDTGRKGGGGKESGGRFGHFPERPFRPKPSASVGFFTARPNGDPTMKQRAFDKPHVTYTIQHNDIEQAHASPRLNMTFLVSGATGLLLLVLIVFLILIVVVRKSTSSKPPPVKSARNRNLRPYSTGTLSKSDIVVLPVPTSEDEEKDTTKPVGADGITTVRLISLEKAATLDKPKSYSTHQDFMAQLKGTLRSRNTYANFFENEAVTKKFDRSNSNSSDFSEGEIVRCDSAFCDDFKPGLSGSETAINDYSPEARFFKDNKTDKSNSTGNLVYVELDFGKAKPTSAVKGGDDKTHYAEIRLSKNIDL
